# Use of genetic variation to separate the effects of early and later life adiposity on disease risk: mendelian randomisation study

**DOI:** 10.1136/bmj.m1203

**Published:** 2020-05-06

**Authors:** Tom G Richardson, Eleanor Sanderson, Benjamin Elsworth, Kate Tilling, George Davey Smith

**Affiliations:** 1 MRC Integrative Epidemiology Unit (IEU), Population Health Sciences, Bristol Medical School, University of Bristol, Oakfield House, Oakfield Grove, Bristol, BS8 2BN, UK

## Abstract

**Objective:**

To evaluate whether body size in early life has an independent effect on risk of disease in later life or whether its influence is mediated by body size in adulthood.

**Design:**

Two sample univariable and multivariable mendelian randomisation.

**Setting:**

The UK Biobank prospective cohort study and four large scale genome-wide association studies (GWAS) consortiums.

**Participants:**

453 169 participants enrolled in UK Biobank and a combined total of more than 700 000 people from different GWAS consortiums.

**Exposures:**

Measured body mass index during adulthood (mean age 56.5) and self-reported perceived body size at age 10.

**Main outcome measures:**

Coronary artery disease, type 2 diabetes, breast cancer, and prostate cancer.

**Results:**

Having a larger genetically predicted body size in early life was associated with an increased odds of coronary artery disease (odds ratio 1.49 for each change in body size category unless stated otherwise, 95% confidence interval 1.33 to 1.68) and type 2 diabetes (2.32, 1.76 to 3.05) based on univariable mendelian randomisation analyses. However, little evidence was found of a direct effect (ie, not through adult body size) based on multivariable mendelian randomisation estimates (coronary artery disease: 1.02, 0.86 to 1.22; type 2 diabetes:1.16, 0.74 to 1.82). In the multivariable mendelian randomisation analysis of breast cancer risk, strong evidence was found of a protective direct effect for larger body size in early life (0.59, 0.50 to 0.71), with less evidence of a direct effect of adult body size on this outcome (1.08, 0.93 to 1.27). Including age at menarche as an additional exposure provided weak evidence of a total causal effect (univariable mendelian randomisation odds ratio 0.98, 95% confidence interval 0.91 to 1.06) but strong evidence of a direct causal effect, independent of early life and adult body size (multivariable mendelian randomisation odds ratio 0.90, 0.85 to 0.95). No strong evidence was found of a causal effect of either early or later life measures on prostate cancer (early life body size odds ratio 1.06, 95% confidence interval 0.81 to 1.40; adult body size 0.87, 0.70 to 1.08).

**Conclusions:**

The findings suggest that the positive association between body size in childhood and risk of coronary artery disease and type 2 diabetes in adulthood can be attributed to individuals remaining large into later life. However, having a smaller body size during childhood might increase the risk of breast cancer regardless of body size in adulthood, with timing of puberty also putatively playing a role.

## Introduction

Obesity in children is widely recognised as a global public health crisis, yet its prevalence continues to rise.^1^ ^2^ Having a high body mass index (BMI) in early life is thought to increase the risk of various health conditions, such as coronary artery disease, type 2 diabetes, and different types of cancer, in later life.^3^ ^4^ ^5^ ^6^ ^7^ Whether an individual can reverse the impact of childhood obesity through lifestyle modifications is unclear, particularly as those who are obese in early life tend to remain obese as adults.^8^ This makes it challenging to discern whether early life adiposity has an independent and lasting influence on disease risk or if its effect is entirely mediated by later life adiposity. If the latter is the case, then the potential adverse consequences of childhood obesity could be avoided by attaining and maintaining a healthy weight in adulthood.

Mendelian randomisation is an approach that can help deal with these challenges^9^ through the use of genetic variants as instrumental variables to infer causality among correlated traits.^10^ ^11^ As an individual’s genotype is established at zygote formation then genetic variation is robust to reverse causation and confounding is considerably less evident than in conventional observational studies.^12^ Furthermore, recent methods have been developed to determine whether several exposures influence an outcome along the same causal pathway or whether the exposures have independent effects.^13^ ^14^ One of these developments is known as multivariable mendelian randomisation.^13^ ^15^

In this mendelian randomisation study we evaluated whether genetically predicted early life body size has an effect on four disease outcomes that have been linked to childhood adiposity: coronary artery disease, type 2 diabetes, breast cancer, and prostate cancer.^3^ ^4^ ^7^ ^16^ We identified genetic instruments by undertaking a genome-wide association study of 453 169 participants in the UK Biobank study with measures of BMI in adulthood (mean age 56.5) and who self-reported perceived body size at age 10 years. This allowed us to conduct both univariable and multivariable mendelian randomisation analyses to discern whether the predicted causal influence of early life body size has an independent effect on disease risk, or whether the effect is mediated through later life body size.

**Fig 1:**
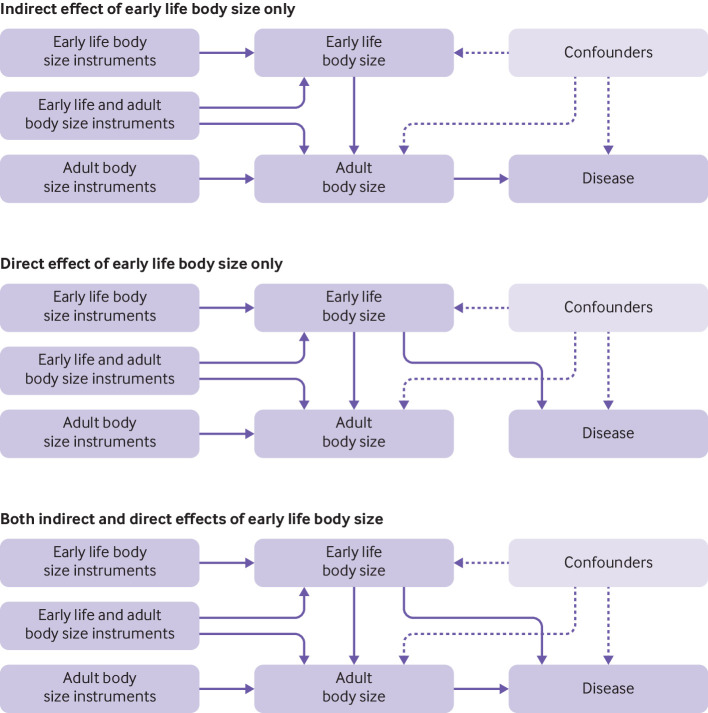
Directed acyclic graphs depicting three possible scenarios that could explain a causal effect between body size at age 10 years and disease outcomes in adulthood. (Top) Early life body size has an indirect effect on disease risk only through body size in adulthood, (middle) early life body size has a direct effect on disease risk independent of body size in adulthood, and (bottom) early life body size exerts both direct and indirect effects on disease risk in adulthood

We postulated that if body size in early life has a causal influence on disease risk, then there are likely three main categories that such effects could be grouped into in a multivariable framework after accounting for body size during adulthood (fig 1). Firstly, the influence of early life body size on disease risk could be mediated by later life body size. As such, early life body size only has an indirect effect on disease risk through adult body size, which, for example, could be attributed to those with a large body size at age 10 remaining overweight into adulthood (fig 1 top panel). Secondly, early life body size might have only an independent (ie, direct) effect on disease risk, which is not mediated through adult body size (fig 1 middle panel). Thirdly, body size at separate time points might influence disease risk through alternate causal pathways (ie, early life body size has both a direct and an indirect effect on outcome; fig 1 bottom panel). These scenarios are possible to investigate using multivariable mendelian randomisation given that a genome-wide association study has recently shown that genetic variation might have varying effects on body size at different stages in the life course.^17^ Supplementary figure 1 provides additional descriptions of direct, indirect, and total effects within a multivariable framework.

## Methods

### UK Biobank and disease outcome datasets

Between 2006 and 2010 the UK Biobank study enrolled 500 000 adults aged between 40 and 69 years at baseline across 22 assessments centres in the United Kingdom.^18^ Data were collected based on clinical examinations, assays of biological samples, detailed information on self-reported health characteristics, and genome-wide genotyping.^19^ BMI was derived using height (measured in whole centimetres) and weight (to the nearest 0.1 kg) measured at baseline. Participants were also asked “When you were 10 years old, compared to average would you describe yourself as thinner, plumper, or about average?” This measure is referred to as early life body size. We performed validation and simulation analyses to account for possible limitations of using perceived body size rather than a measured variable. Only those with measures in both early life and later life were included in analyses. BMI in adults was converted into a categorical variable with three groups based on the same proportions as the early life body size variable (ie, thinner, plumper, and about average). This was to ensure that both measures were as comparable as possible. This measure is referred to as adult body size. Effect estimates from our results can be interpreted as the increase in odds conferred for each additive change in body size category. All individual participant data used in this study were obtained from the UK Biobank study. Participants enrolled in UK Biobank have signed consent forms.

In total, 12 370 749 genetic variants in 463 005 people were available for analysis, as described previously.^20^ Briefly, UK Biobank participants were selected based on those of European descent (using K means clustering (K=4)) after standard exclusions, including withdrawn consent, mismatch between genetic and reported sex, and putative sex chromosome aneuploidy.^21^ Effect estimates for genome-wide genetic variants on coronary artery disease, type 2 diabetes, breast cancer, and prostate cancer were obtained using findings from large scale consortiums, which did not include data from the UK Biobank (supplementary table 1).^22^ ^23^ ^24^ ^25^ Since earlier age at menarche is an established risk factor for breast cancer we also obtained summary statistics from a genome-wide association study of age at menarche that did not include individual’s from the UK Biobank, which was used in additional analyses relating to this outcome.^26^

### Avon Longitudinal Study of Parents and Children

The Avon Longitudinal Study of Parents and Children (ALSPAC) is a population based cohort study investigating genetic and environmental factors that affect the health and development of children. The study methods are described in detail elsewhere.^27^ ^28^ Briefly, 14 541 pregnant women residing in the former region of Avon, UK, with an expected delivery date between 1 April 1991 and 31 December 1992 were eligible to take part in ALSPAC. Detailed phenotypic information, biological samples, and genetic data have been collected from the participants, which are available through a searchable data dictionary (http://www.bristol.ac.uk/alspac/researchers/our-data/). Written informed consent was obtained for all study participants. Ethical approval for the study was obtained from the ALSPAC ethics and law committee and the local research ethics committees. Supplementary note 1 provides further details on genotyping and trait measurements in the ALSPAC cohort.

### Statistical analysis

#### Identifying instruments

We used the software BOLT-LMM to assess the association between genetic variants across the human genome and both measures of body size.^20^ ^29^ This approach applies a bayesian linear mixed model to evaluate the association between each genetic variant with each measure of body size in turn, while accounting for both relatedness and population stratification. We added age at baseline and type of genotyping array as covariates in the model. Analyses were undertaken three times, once in all eligible participants after additionally adjusting for sex, and then stratifying by sex and analysing each separately. This allowed us to identify genetic variants that could be used as instrumental variables for outcomes based on populations of women only (ie, breast cancer), populations of men only (ie, prostate cancer), and mixed populations (ie, coronary artery disease and type 2 diabetes). Analyses of early life body size were additionally adjusted for month of birth as we hypothesised that peoples’ relative age within their school year might bias self-reporting. Effect estimates from this analysis were used in the subsequent mendelian randomisation analyses. We used linear regression—that is, assuming that the effect of a given single nucleotide polymorphism on moving from the lowest to the middle category of the body size variables is the same as its effect on moving from the middle to the highest.

We used bivariate GREML analysis with GCTA software to calculate the genetic correlation between the early life and adult body size results from a genome-wide association study.^30^ A genetic relation matrix was derived from 10 000 randomly selected UK Biobank participants who were unrelated and of European descent. To identify independent variants from our genome-wide association study we undertook linkage disequilibrium clumping using the software PLINK.^31^ This was based on a threshold of r^2^<0.001 using genotype data from European individuals from phase 3 (version 5) enrolled in the 1000 genomes project as a reference panel.^32^ For independent variants associated with either early life or adult body size in our genome-wide association study of all participants (based on P<5×10^−08^), we compared the effect estimates of these variants between time points. This was undertaken by stacking observations (ie, so that each individual had two body size measurements) and then regressing the categorical body size outcomes on each variant in turn adjusting for age, sex, and time (ie, early life or later life). This analysis was then repeated along with adjustment for the interaction between genetic variant and time period. To account for multiple testing, we applied a Bonferroni correction to the P values of the interaction terms (ie, P<0.05/number of individual variants assessed) as a heuristic to highlight genetic loci with the strongest evidence of an interaction with time. This analysis was undertaken to show that various genetic variants seemingly appear to have a stronger influence on body size at different time points in the life course.

#### Validation of genetic scores using external datasets

Data on clinically measured BMI from the ALSPAC cohort was used to validate the scores we generated in the UK Biobank study. BMI data were obtained at three time points; two based on the ALSPAC offspring (at mean ages 9.9 and 17.9 years) and one in the ALSPAC mothers (mean age 50.8 years). Measures of BMI were dichotomised to classify individuals higher than the 85th centiles as overweight. Receiver operator characteristic curves were generated using the R package plotROC to compare the predictive ability of the scores for both early life and adult body size at all three time points.

We also undertook linkage disequilibrium score regression^33^ to evaluate the genetic correlation between the two measures of body size from our analysis with directly measured BMI in adulthood,^34^ and childhood obesity^35^ using large scale external populations based on data from the linkage disequilibrium hub.^36^ This was undertaken to further validate our early life measure of body size by showing that it is more strongly correlated with measured obesity in childhood than with BMI in adulthood. This was particularly important to show given that the early life body size score in UK Biobank was derived using questionnaire recall data, which could lead to bias if not appropriately validated using external datasets.

#### Mendelian randomisation

Univariable mendelian randomisation analyses were conducted using the MR-Base platform^37^ to investigate total effect estimates between genetically predicted early life and adult body size individually with each disease outcome in turn. This was based on the inverse variance weighted method, which estimates the causal effect of an exposure on an outcome by combining ratio estimates using each variant in a fixed effect meta-analysis model.^38^ Effect estimates on early life body size were used for the analysis, with additional adjustment for month of birth. However, the “total” effect of adult body size should be interpreted with caution. A strong correlation is likely to exist between genetic determinants of early and adult body size, and thus the total effect of adult body size is likely to be driven by pleiotropy if there is also a direct effect of early body size on the outcome. For this reason we only discuss the total effect of early body size and the direct effects of early and adult body size. Supplementary figure 1 shows a direct acyclic graph illustrating direct, indirect, and total effects as investigated within a multivariable mendelian randomisation framework.

We undertook multivariable mendelian randomisation analyses to estimate the direct effect of early life body size on each outcome in turn. Variants from the univariable analysis were used again here after undertaking further linkage disequilibrium clumping to account for instrument correlation between the two sets. We also undertook a negative control analysis with early life and adult body size as exposures in a multivariable framework and age at menarche as an outcome using findings from a genome-wide association study published before the release of UK Biobank.^26^ We hypothesised that early life body size should only influence age at menarche directly in this framework as an individual’s adult body size cannot influence timing of puberty (supplementary figure 2). As such, direct and total effects derived from a univariable framework should be comparable for early life body size on this outcome, whereas evidence of an indirect effect (ie, through adult body size) should be weak.

Furthermore, we undertook a sensitivity analysis for the breast cancer outcome to discern whether timing of puberty might also play a role in disease risk. This involved repeating analyses on breast cancer with age at menarche as a third exposure (along with early life and adult body size). We also repeated the univariable and multivariable mendelian randomisation analyses using findings derived from women with oestrogen receptor positive and oestrogen receptor negative breast cancer. Moreover, we undertook various sensitivity analyses to investigate results from the univariable and multivariable analyses, such as assessing instrument strength by deriving F statistics,^39^ examining heterogeneity using Cochran’s Q,^40^ and investigating horizontal pleiotropy using the MR-Egger method.^41^ ^42^ Finally, we undertook a simulation study to investigate how misclassification of early life body size could affect our results (supplementary note 2).

All analyses were undertaken using R (version 3.5.1). Plots were created using the packages ggplot2^43^ and metaphor.^44^

### Patient and public involvement

This research did not involve patients or the public as it uses data from the UK Biobank study that were previously obtained from a cohort of people who had already been recruited. As such, no patients or member of the public were involved in the design or implementation of this study or the research questions addressed.

## Results

**Fig 2:**
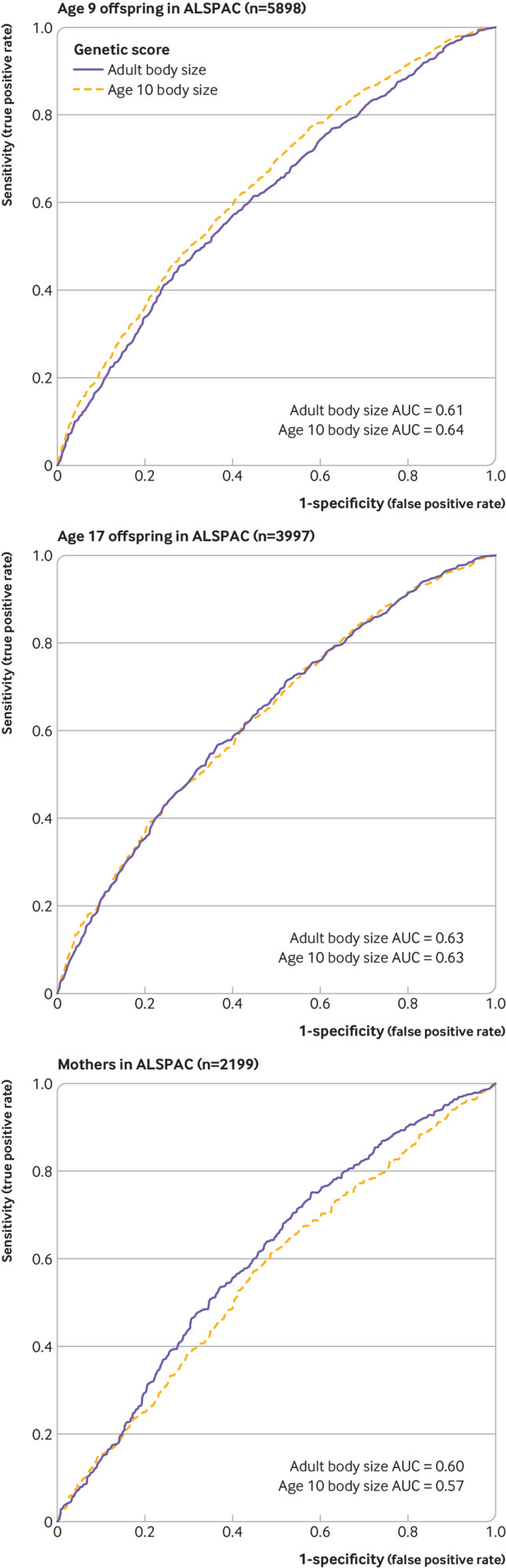
Receiver operator characteristic curves to compare the predictive capability of early life and adult body size scores across three time points in Avon Longitudinal Study of Children and Parents (ALSPAC). (Top) Mean age 9.9 years in the ALSPSAC offspring; (middle) mean age 17.9 years in the ALSPAC offspring; and (bottom) mean age 50.8 years in the ALSPAC mothers. Body mass index in the ALSPAC cohort was dichotomised based on the 85th centile in all analyses. AUC=area under curve

In a genome-wide association study of 453 169 UK Biobank participants, 295 and 557 independent associations were detected with early life and adult body size, respectively, based on conventional genome-wide corrections (ie, P<5×10^−08^; supplementary tables 2 and 3). A genetic correlation coefficient of rG=0.61 was calculated between these two sets of results. Using individual level data from the ALSPAC cohort, a genetic score based on the early life body size variants was found to be a stronger predictor of childhood BMI (mean age 9.9 years) compared with a score based on the adult variants (fig 2 left panel). Investigating the prediction of both early life and adult body size scores during later adolescence (mean age 17.8 years) suggested that neither score was optimal at this time point (fig 2 middle panel). During the adulthood time point (mean age 50.8 years), the adult body size score was a better predictor than using the early life body size variants (fig 2 right panel). These findings show the ability of these genetic instruments to separate early life size from adult body size.

Linkage disequilibrium score regression analyses identified strong genetic correlation between our derived early life measure of body size and a previous genome-wide association study of childhood obesity (rg=0.85) (supplementary figure 3). In contrast, our adult body size measure was considerably more strongly genetically correlated with a previous genome-wide association study of BMI in adulthood (rg=0.96) than to obesity in childhood (rg=0.64). Evaluating the difference between the genetic variant associations at the two different time points suggested that 75 genetic variants had stronger effect estimates on body size in adulthood compared with body size in early life, whereas 23 genetic variants had a stronger effect in early life compared with adult body size estimates (at P<7.19×10^−05^ (ie, 0.05/694 tests), supplementary table 4). Demonstrating that the magnitude of effect for these genetic variants differs with respect to early life and adult body size further suggests that they can be separated as two exposures in a multivariable framework. Plotting the relation between early life body size and adult body size in UK Biobank did not indicate a non-linear relation between them in line with the assumptions of multivariable mendelian randomisation (supplementary figure 4). Linear trajectories between childhood and adulthood have also been presented previously in cohorts, such as the Fels Longitudinal Study.^45^

**Fig 3:**
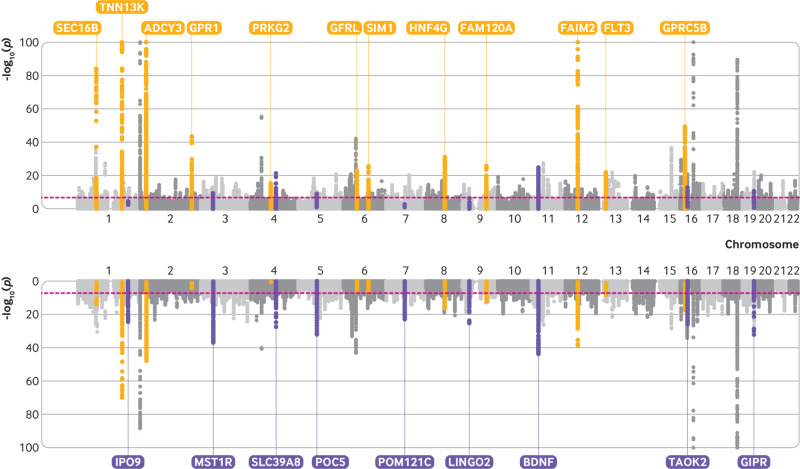
Bidirectional Manhattan plot to illustrate genetic variants across the genome associated with (top) body size in early life (age 10 years) and body size in adulthood (bottom). Yellow points highlight effects that provided stronger evidence of association with early life body size, whereas purple points highlight effects more strongly associated with adult body size. These genetic loci have been annotated based on the nearest protein coding gene to the top associated variant

Figure 3 illustrates findings from both early life and adult body size genome-wide association studies using a bidirectional Manhattan plot (sometimes referred to as a Miami plot) where some examples of time point specific effects have been highlighted. Repeating our genome-wide association study stratified by sex identified 135 and 215 variants that survived genome-wide association study corrections (ie, P<5×10^−08^) in women only with early life and adult body size, respectively (n=246 511) (supplementary tables 5 and 6). In the analysis restricted to men only, 69 genetic variants were associated with early life body size and 159 genetic variants were associated with adult body size (n=206 658) (supplementary tables 7 and 8). Repeating early life body size analyses with additional adjustment for month of birth did not seem to materially influence overall findings (supplementary tables 9-11).

### Univariable and multivariable mendelian randomisation analyses

**Fig 4:**
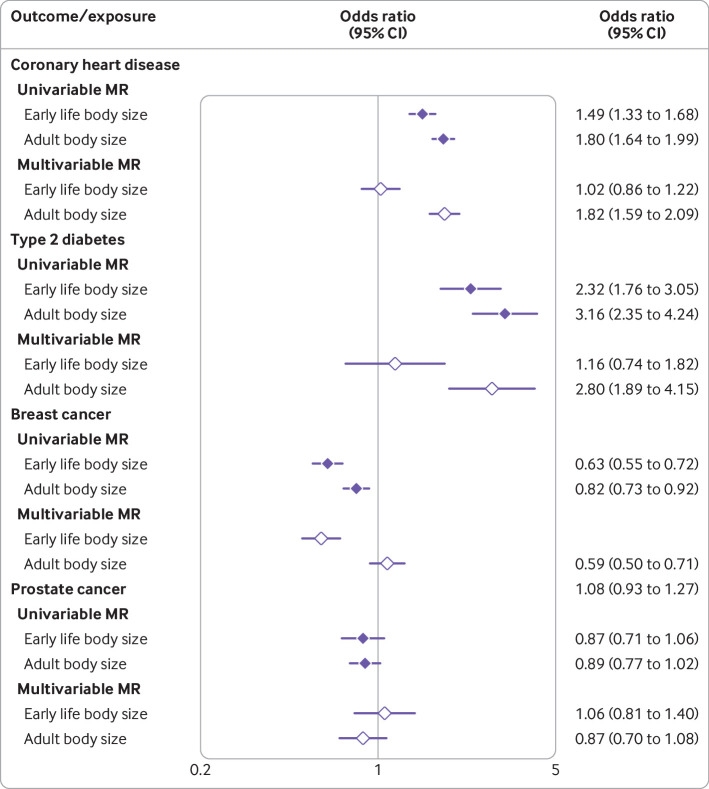
Forest plot illustrating direct and indirect effects for genetically predicted body size in early life (age 10 years) on four different disease outcomes (coronary artery disease, type 2 diabetes, breast cancer, and prostate cancer). Filled and open diamonds represent effects from univariable and multivariable mendelian randomisation (MR) analyses, respectively. Estimates are displayed as odds ratios with 95% confidence intervals

Odds ratios from these analyses reflect the change in odds for each change in category for our derived early life and adult body size variables. Univariable analyses provided strong evidence that early life body size is associated with risk of coronary artery disease (univariable mendelian randomisation odds ratio 1.49, 95% confidence interval 1.33 to 1.68). However, the direct effect of early life body size (ie, not mediated through adult body size) was much smaller than the total effect (multivariable mendelian randomisation odds ratio 1.02, 95% confidence interval 0.86 to 1.22), whereas strong evidence of a direct effect was identified for adult body size (multivariable mendelian randomisation odds ratio 1.82, 1.59 to 2.09). Similar findings were identified for analyses of type 2 diabetes, as the magnitude of the direct causal effect for early life body size was much smaller than the total effect (multivariable mendelian randomisation odds ratio: early life body size 1.16, 0.74 to 1.82, adult body size 2.80, 1.89 to 4.15). Figure 4 shows effect estimates from both univariable and multivariable mendelian randomisation analyses (supplementary table 12 also provides a full list). The results from this analysis were robust to the various sensitivity analyses applied in this study (supplementary tables 13-15).

The univariable analysis evaluating the effect of early life body size on age at menarche as an outcome provided strong evidence that having a larger body size at age 10 might result in earlier puberty (β −0.93 standard deviation change in age at menarche for each change in body size category, 95% confidence interval −0.66 to −1.20). When adult body size was included in the multivariable framework, along with early life body size as an exposure, the direct effect estimate for early life body size was found to be essentially unchanged (table 1). In contrast, evidence that adult body size has an effect on timing of puberty was weak. These findings reflect the scenario depicted in supplementary figure 2, suggesting that early life body size can only affect timing of puberty directly and not indirectly through adult body size. This analysis serves as a proof of concept for our multivariable framework, given that adult body size cannot influence timing of puberty as it occurs at a later time point in the life course.

**Table 1:** Estimates from univariable and multivariable mendelian randomisation analysis assessing effect of predicted early life and adult body size on age at menarche, and sensitivity analysis to investigate how both these exposures influence breast cancer risk when modelled with age at menarche as an additional third exposure

Outcomes and exposures	No of SNPs*	Univariable analysis			Multivariable analysis	
		β† (SE)	P value		β† (SE)	P value
Age at menarche^26^:						
Early life body size	102	−0.93 (0.14)	1.42×10^−10^		−0.94 (0.15)	3.87×10^−10^
Adult body size	142	-	-		−0.03 (0.14)	0.82
Breast cancer^24^:						
Early life body size	124	−0.46 (0.07)	3.84×10^−11^		−0.64 (0.10)	1.57×10^−10^
Adult body size	191	-	-		0.06 (0.09)	0.52
Age at menarche	60	−0.02 (0.04)	0.58		−0.10 (0.03)	4.84×10^−03^

SNPs=single nucleotide polymorphisms.

* Number used as instrumental variables.

† Effect estimate coefficient for each standardised unit change in exposure.

In the univariable analysis strong evidence was found that higher genetically predicted early life body size was associated with a reduction in risk of breast cancer (univariable mendelian randomisation odds ratio 0.63, 0.55 to 0.72). When both measures of body size were analysed in a multivariable framework, the direct effect of adult body size (multivariable mendelian randomisation odds ratio 1.08, 0.93 to 1.27) was smaller in magnitude than the direct protective effect of early life body size (0.59, 0.50 to 0.71). Similar findings were identified in the analysis of women with oestrogen receptor positive and oestrogen receptor negative breast cancer (supplementary table 16).

Evaluating the relation between genetically predicted age at menarche and risk of breast cancer in a univariable analysis provided weak evidence of a total effect (univariable mendelian randomisation odds ratio 0.98, 0.90 to 1.06). However, when modelled together with both early and adult body size (ie, all three exposures in the multivariable model) there was strong evidence that later age at menarche might lower the risk of breast cancer (multivariable mendelian randomisation odds ratio 0.90, 0.85 to 0.95). Effect estimates for predicted early life body size had a larger effect in this multivariable analysis compared with the direct effect of adult body size (table 1). Evidence that early life body size has a predicted causal effect on prostate cancer directly was weak (multivariable mendelian randomisation odds ratio 1.06, 0.81 to 1.40), which was also the case for adult body size (0.87, 0.70 to 1.08).

Early life body size was self-reported later in life and so is prone to misclassification, whereas adult body size is directly measured and is likely to be subject only to random error. A simulation was therefore conducted to identify how such misclassification, affecting only one exposure, could influence the effect estimation in the multivariable mendelian randomisation analyses. The simulation study (see supplementary note 2) shows that misclassification of early life body size is associated with a weakening in strength of association between the single nucleotide polymorphisms and our measure of early life body size and so potentially biases the estimated effect of early life body size in the multivariable mendelian randomisation analysis. These simulations do not cover all of the potential scenarios for measurement error. In the scenarios considered, however, misclassification of early life body size only introduces bias in the estimated effect of adult body size on the outcome when the misclassification in early life body size is dependent on actual adult body size and there is an effect of early life body size on the outcome. The simulations also show that the direction of this bias on the estimated effect of the adult variable depends on the direction of effect of the adult body size relative to the early life measure. Furthermore, the effect of adult body size is unbiased when early life body size has no associated effect on the outcome.

The simulation study also shows that the potential measurement error in self-reported body size at age 10 has the potential to bias adult effects in the multivariable mendelian randomisation when the measurement error depends on observed adult BMI and early life body size has a causal effect on the outcome. This therefore has the potential to affect the results we obtain for adult body size and risk of breast cancer. These simulations show that the bias from misclassification depends on both the type of misclassification and the size and direction of the effects of both exposures on the outcome. However, they suggest that this misclassification only masks the adult effect when it acts in the same direction as the early life effect on the outcome. We therefore do not believe that this measurement error is hiding a risk increasing effect of adult body size on breast cancer, although it is possible that it is masking a larger protective effect.

## Discussion

In this study we examined the influence of body size in early life (age 10 years) on risk of disease in later life and whether this putative causal effect occurs independently (direct effect) or through the same causal pathway (indirect effect) as later life body size. Our univariable mendelian randomisation analysis suggested that genetically predicted early life body size is associated with an increased risk of coronary artery disease and type 2 diabetes. When early life body size was analysed together with adult body size in a multivariable framework, however, the direct effect estimates for early life body size were considerably attenuated and fully compatible with, and close to, the null compared with the estimates of the total effects, suggesting that the influences of early life adiposity on these outcomes are mediated by body size in later life (see fig 1). These findings imply that observed associations between early life obesity and increased risk of coronary artery disease and type 2 diabetes are likely attributed to those with a large body size in childhood that persists into later life. This suggests that a window of opportunity exists to mitigate the detrimental impact of early life body size on risk of these disease outcomes.^46^ ^47^ These findings corroborate those from previous studies suggesting that there is no persistent influence of childhood obesity on risk of type 2 diabetes and cardiovascular disease unless adiposity is sustained.^48^ ^49^ Furthermore, these findings highlight the importance of taking into account adult body size to assess whether childhood body size has a direct or persistent effect on disease risk over the life course.^50^ ^51^

Our results also provide strong evidence that early life body size has a protective effect on risk of breast cancer, as has been previously reported from both observational and mendelian randomisation studies that have not taken into account later life body size.^52^ ^53^ ^54^ ^55^ This was identified as both a total and a direct effect using univariable and multivariable mendelian randomisation analyses, respectively, suggesting that the effect of early life body size might persist into later life regardless of interventions that influence variation in body size. Furthermore, reported protective effect estimates from mendelian randomisation studies between later life body size and breast cancer risk could be attributed to effects from childhood. This is in contrast to observational estimates suggesting that higher BMI in adulthood might increase the risk of breast cancer, which could be attributed to confounding factors to which mendelian randomisation analyses are more robust.^55^ This effect requires further investigation in subpopulations of premenopausal and postmenopausal women with breast cancer from large scale genome-wide association studies. This is particularly crucial given observational evidence suggesting that higher BMI increases the risk of invasive breast cancer in postmenopausal women.^56^

As an additional test of our multivariable framework, we undertook a negative control analysis to investigate the effect of early life body size on age at menarche in both a univariable and a multivariable framework accounting for adult body size. We found that estimates were consistent from both analyses for early life body size, whereas the effect estimates for adult body size in the multivariable analyses had a much smaller magnitude of effect in comparison. This provides a powerful proof of concept for our analytical framework, given that body size can only influence timing of puberty in early life whereas adult body size cannot as it occurs earlier in the life course. Moreover, our results support findings from the literature suggesting that higher BMI in childhood can lead to earlier timing of puberty.^57^ Evidence is particularly strong in women, where an evolutionary mechanism rendering adequate fat storage to sustain both mother and growing fetus has been postulated.^58^

We undertook an additional analysis to investigate timing of puberty with respect to the putative protective effect of early life body size on risk of breast cancer. To do this we incorporated age at menarche as an additional exposure in our multivariable mendelian randomisation analysis. Our findings corroborate similar analyses undertaken using a multivariable framework, which suggest that later age at menarche has a protective effect on breast cancer risk but only when accounting for early life body size.^59^ Evidence from the literature of a relation between timing of puberty and later life BMI is, however, strong.^60^ ^61^ Notably, our results build on previous findings by modelling early life and adult body size as two separate exposures, with the direct effect of early life body size providing strong evidence of a protective effect when accounting for age at menarche. Similar observational findings have been reported recently in premenopausal women after adjusting for age at menarche.^62^ Developing insight into the underlying biological mechanisms explaining this effect could highlight potentially modifiable pathways. In terms of proposed explanations, the earlier pubertal onset attributed to higher childhood adiposity has been postulated to result in slower pubertal growth.^63^ This might therefore protect against rapid pubertal growth during adolescence, which has been linked to an increased risk of breast cancer.^64^ Hormonal mechanisms might also play a role, such as higher oestrogen levels in early life produced by an increase in adipose tissue.^62^ Oestrogenic effects have been reported to induce breast differentiation in early life, as well as to increase the expression of tumour suppressor genes.^65^

We were unable to support previous evidence of an effect between genetically predicted body size and prostate cancer risk.^66^ ^67^ This relation is complex owing to the effect of obesity on various hormones in men, such as an inverse relation with prostate specific antigen^68^ and a positive relation with oestrogen concentrations.^69^ This outcome is therefore worth revisiting using this study’s analytical framework when findings from a forthcoming large scale genome-wide association study of prostate cancer become available. A greater number of genetic instruments in men only is also likely to improve power for future endeavours. Evaluating the influence of pubertal development on this outcome would also be a worthwhile undertaking.^70^

We used univariable mendelian randomisation to estimate total effects of early body size, and multivariable mendelian randomisation to estimate direct effects of early and adult body size. In theory, the univariable estimate of the effect of adult body size (the total effect) and the multivariable estimate (the direct effect) should be equal. However, if some genetic variants influence both early and adult body size (albeit with different effect sizes), and early life body size has a direct effect on the outcome, then this will potentially generate bias in the univariable estimate of adult body size. This can be seen in the univariable estimate of the effect of adult body size on breast cancer risk, which is quite different to the multivariable estimate, probably related to the direct effect of early body size on breast cancer risk. For coronary artery disease, where there is no direct effect of early body size on the outcome, the univariable and multivariable effects of adult body size are the same. This highlights one of the problems with mendelian randomisation of time varying exposures—the univariable analyses cannot identify critical or sensitive periods of exposure but only an effect of a difference in the cumulative lifetime exposure.^9^ ^71^ ^72^ ^73^ Moreover, we assume that childhood body size has an effect on adulthood body size in all scenarios (see fig 1). Therefore, those who, for example, reduce their body size between childhood and adulthood might effectively break this mediated causal chain and reduce their increased risk for diseases such as coronary artery disease or type 2 diabetes.

### Strengths and limitations of this study

The key strengths of our investigation include the large number of participants from the UK Biobank study with measures of both early life and later life adiposity (n=453 169). Although this sample size is large, a caveat is reliance on retrospective questionnaire based data for the early life variable. Our early life variable is therefore based on perceived early life body size, which also meant that for harmonisation purposes we had to generate a similarly categorised adult variable. As such there will likely be additional measurement error in the early life variable, which could be differential with respect to adult body size (ie, larger adults might misremember their size at age 10 differently from those who are thinner). The statistical power in our genome-wide association study therefore comes at a cost, although s we were able to recapitulate evidence of association between genetic variants and early life body size measurements in the literature.^74^ ^75^ Furthermore, we were able to validate our scores using individual level data from the ALSPAC cohort, showing that the score in early life was a superior predictor of childhood adiposity, whereas the score in adulthood performed better in adults. Along with the large sample size in UK Biobank, other benefits of this approach included being able to harmonise our early life and adult measures within the same sample of participants. This was undertaken to reduce the likelihood of bias from differing samples—for example, between two separate genome-wide association study consortiums involving differing populations. Moreover, measurements of BMI in childhood taken from current generations might not reflect those of older populations that contributed to the outcome estimates in our analysis, whereas the UK Biobank participants and the outcome genome-wide association studies are from similar birth cohorts.

Another strength of this study was that we used two sample mendelian randomisation to harness large scale summary statistics from genome-wide association studies. This circumvents the necessity of having both exposures and outcomes measured in the same sample. Moreover, along with using genetic variants to mitigate the influence of confounding and reverse causation, a multivariable mendelian randomisation framework enabled us to investigate the independent effect of early life body size, which is extremely challenging in an observational setting. This framework therefore presents a powerful means to investigate many other questions in epidemiology. These could be related to the effect of the same exposure at different time points in the life course, as investigated in this study of body size, or examining whether different risk factors influence disease risk independently or along the same causal pathway. Particularly given the large scale summary level data, which current health data researchers are fortunate to have access to, this presents an attractive opportunity to analyse the life course structure of the causes of disease using mendelian randomisation.

Our univariable analysis used all the single nucleotide polymorphisms associated with each exposure. Although there are variations in the single nucleotide polymorphisms associated with each of early life and adult BMI, considerable overlap also exists in the single nucleotide polymorphisms associated with each time point. Our univariable mendelian randomisation analyses therefore capture pleiotropic effects of the single nucleotide polymorphisms through the other time point as well as, for childhood BMI, the effect mediated by adult body size. This is a limitation of our univariable mendelian randomisation analyses but highlights the importance of the multivariable mendelian randomisation to disentangle the direct effects of child and adult body size. It should also be emphasised that we have used genetically determined body size as exposure in this work, which might not directly equate to weight loss or gain from lifestyle modifications. Moreover, we acknowledge that survival bias can distort findings from mendelian randomisation analyses, as discussed in a recent study.^76^ Although this is more likely to be a problem for outcomes that typically have a later onset of disease than those studied in this work (eg, Alzheimer’s disease), replication of our results is necessary to rule out the possibility of survival bias.

Another limitation of our study is that participants from the UK Biobank could have also contributed to the large scale genome-wide association study, the results of which were used in this study. Overlapping exposure and outcome samples in a two sample mendelian randomisation analysis might result in overfitting, although currently there is no way to discern whether this is the case unless anonymous identifiers between the UK Biobank and genome-wide association studies consortium can be linked together.^77^ Moreover, estimates used in this study are based solely on body size and BMI data from the UK Biobank. These findings should therefore be evaluated in future cohorts when sample sizes make this possible. This is particularly important as it has been shown that UK Biobank participants are highly selected, which can be problematic for instrumental variables analyses.^78^ ^79^ However, severe selection bias that can result in reversed estimates is unlikely to be a problem in our study given that the analyses are consistent with previous mendelian randomisation studies.^55^ ^80^ ^81^ Lastly, capturing non-linear effects between body size at different points in the life courses and disease outcomes is challenging in a two sample setting.^82^ Development of methods is therefore warranted.

### Conclusions

Using multivariable mendelian randomisation, we have provided strong evidence suggesting that early life adiposity has a causal influence on risk of breast cancer that acts independently of later life BMI. Conversely, our results suggest that the association between early life adiposity and risk of coronary artery disease and type 2 diabetes is likely due to those with a high BMI remaining overweight in later life. Our approach therefore yields insight into the pathway between early life risk factors such as BMI and disease outcomes. Furthermore, our approach provides scope to differentiate between effects when the conferred risk is or is not reversible by achieving and maintaining a healthy BMI in adulthood.

**What is already known on this topic**

Obesity in childhood is known to have a detrimental impact on various health conditions and disease risk in later life
Body size in early life has been associated with increased odds of coronary artery disease and type 2 diabetes observationally, but whether this effect is immutable or whether lifestyle modifications can help mitigate it is unclear
The influence of early life body size on risk of other diseases, such as breast cancer and prostate cancer, has been previously reported but the causal relations underlying these observations are complex and require further evaluation

**What this study adds**

The influence of genetically predicted early life body size on odds of coronary artery disease and type 2 diabetes was predominantly mediated through later life body size, suggesting that the influence of body size on these outcomes is attributed to a cumulative risk conferred throughout the life course
Findings for body size at age 10 suggested that it had a protective effect on breast cancer risk independent of later life body size, although timing of puberty might play a causal role in this relation
This study focused on cardiometabolic and cancer outcomes, but the analytical approach has potential to investigate how risk factor trajectories across life influence a range of health outcomes

## Web extra

Extra material supplied by authors

**Web appendix:** Supplementary material

**Web appendix:** Supplementary tables

## Notes

Contributors: GDS conceived the study. TGR, ES, KT, and GDS contributed to the study design. TGR conducted the main analysis and wrote the original draft of the manuscript. ES undertook simulation and sensitivity analyses. BE applied genome-wide association studies to derive genetic instruments. All authors were involved in the interpretation of results, helped refine the manuscript, and approved its final version. TGR is the guarantor and attests that all listed authors meet authorship criteria and that no others meeting the criteria have been omitted.

Ethical approval: UK Biobank has received ethical approval from the UK national health service’s National Research Ethics Service (ref 11/NW/0382).

Data sharing: All genetic instruments derived in this study are in the supplementary tables. Individual level data used to derive these results can be obtained with an approved application to the UK Biobank study. Validation data can be obtained with an approved application to the Avon Longitudinal Study of Parents and Children study. Outcome estimates were obtained using publicly accessible data made accessible by genome-wide association study consortiums. Code used to undertake mendelian randomisation analyses can be found as part of the “TwoSampleMR” R package.

The manuscript’s guarantor (TGR) affirms that the manuscript is an honest, accurate, and transparent account of the study being reported; that no important aspects of the study have been omitted; and that any discrepancies from the study as planned (and, if relevant, registered) have been explained.

Dissemination to participants and related patient and public communities: We plan on disseminating the results of this study to any of the study participants or wider relevant communities on request.
