# Supplementary material for: Use of genetic variation to separate the effects of early and later life adiposity on disease risk: mendelian randomisation study
Source: BMJ. 2020 May 6;369:m1203. doi: 10.1136/bmj.m1203 (PMC7201936; doi:10.1136/bmj.m1203)
Supplement: Supplementary file 1 — Web appendix: Supplementary material [file rict052821.ww.pdf]

Supplementary material

Supplementary Figure 1: A direct acyclic graph to demonstrate direct, indirect and total effects estimated within a multivariable Mendelian randomization framework

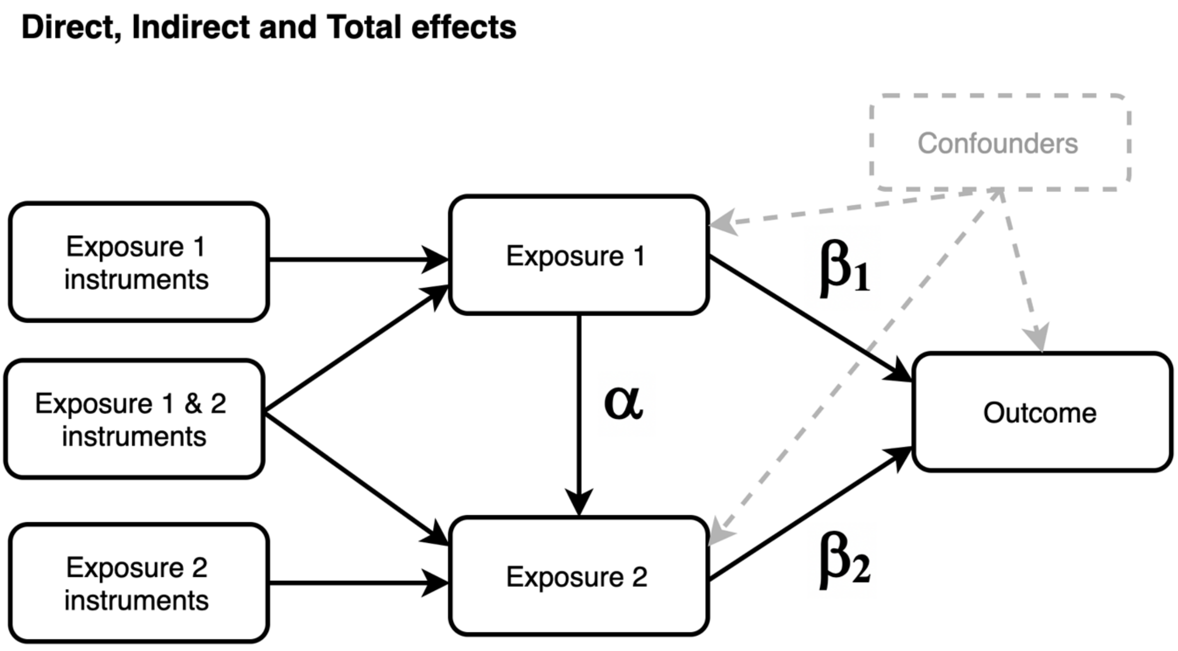

In a multivariable Mendelian randomization framework, the direct effect of exposure 1 on the outcome can be estimated as β_1*, whilst its indirect effect is estimated as* $\alpha\beta_2$*. The total effect is therefore calculated as* $\beta_1 + \alpha\beta_2$*.*

Supplementary Figure 2: An applied negative control example using age at menarche as an outcome

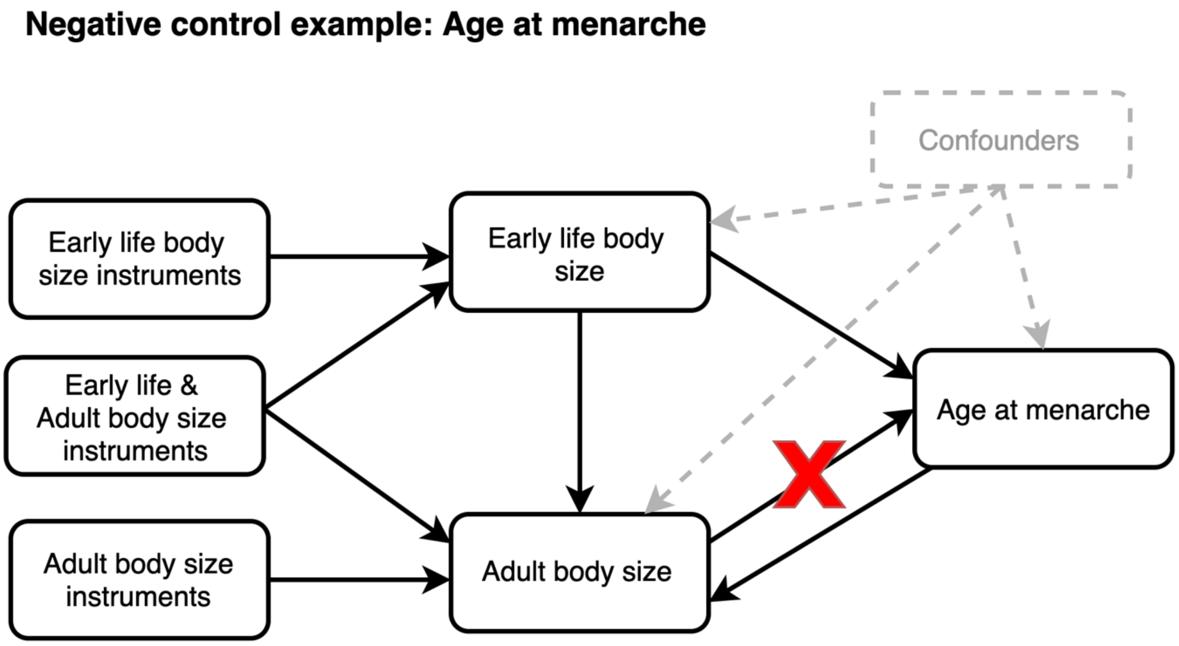

A direct acyclic graph illustrating a multivariable Mendelian randomization (MVMR) analysis to investigate the direct and indirect effect of early life body size on age at menarche. This analysis was undertaken as a negative control, given that early life body size can only influence timing of puberty directly as the indirect path via adult body size is not biologically plausible given that this outcome occurs at an earlier stage in the life course. As such, the direct effect of early life body size as calculated in the MVMR analysis should be the same as the total effect as derived by univariable estimates.

Supplementary Figure 3: Genetic correlation analysis

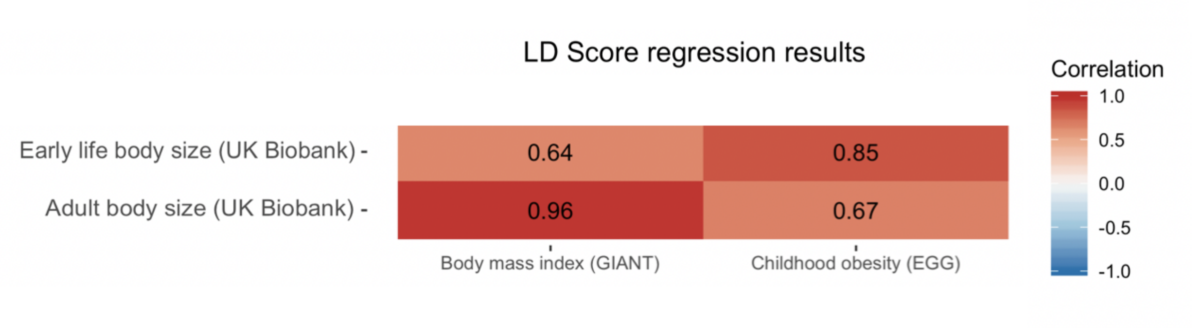

Using linkage disequilibrium (LD) score regression, we compared the genetic correlation between our two exposures (early life and adult body body size) with measured adult body mass index (BMI) and childhood obesity from two consortia (GIANT and EGG).

Supplementary Figure 4: Boxplots for early life and adult body size in UK Biobank

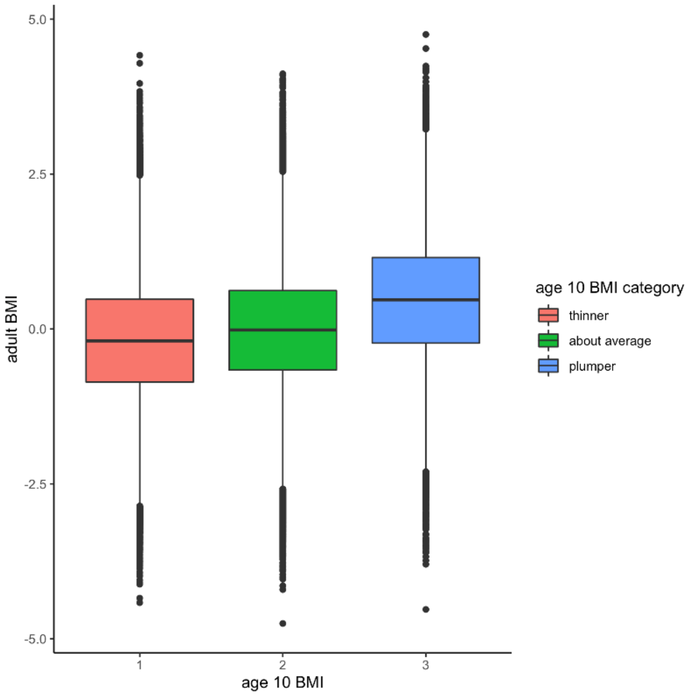

Early life body size is categorised into three groups in the UK Biobank study (based on whether individuals considered themselves to be 'thinner', 'about average' or 'plumper'. Adult body mass index was measured as a continuous variable and normalised to have a mean=1 and standard deviation=0. The categorical measurement for early life BMI made it challenging to investigate the assumption regarding linearity between these exposures in our model. However, these boxplots do not indicate that there was evidence against a linear relationship as there was an overall incremental trend across categories.

Supplementary Note 1: The Avon Longitudinal Study of Parents and Children (ALSPAC) cohort description

All children were genotyped using the Illumina HumanHap550 quad genome-wide SNP genotyping platform. ALSPAC mothers were genotyped using the Illumina human660W-quad array at Centre National de 331 Génotypage (CNG). Genotypes were called with Illumina GenomeStudio. Samples were removed if individuals were related or of non-European genetic ancestry. Genetic variants were removed if they had >5% missingness or a Hardy Weinberg equilibrium (HWE) P-value $<1.0\text{x}10^{-06}$. Imputation was performed using Impute V2.2.2 against a reference panel from the Haplotype Reference Consortium (HRCr1.1, 2016) based on approximately 31,000 phased whole genomes. The HRC panel was phased using ShapeIt v2, and the imputation was performed using the Michigan imputation server. After imputation, we filtered out variants and kept those with an imputation quality score of ≥ 0.8 and minor allele frequency (MAF) > 0.01.

All BMI measurements were obtained at ALSPAC clinics. Height was measured to the nearest 0.1 cm with a Harpenden Stadiometer (Holtain Crosswell), and weight was measured to the nearest 0.1 kg on Tanita electronic scales. Body mass index (BMI) was calculated as (weight [kg]/(height [m]^2).

Supplementary Note 2: Simulation for child and adult BMI estimates

Our measure of early life body size is based on recalled relative body size at age 10, reported by individuals much later in life. This measure is therefore likely to be subject to misclassification due to individuals misremembering their relative body size. Such recall bias will not affect our measure of later life adiposity which is constructed from measures of height and weight taken at the UK Biobank clinic. We therefore computed a simple simulation study to identify what likely effect such misclassification, affecting only one exposure in a two-exposure model, will have on the estimated effects in our multivariable MR estimation.

We set up our model with two positively correlated exposures which each have a causal effect on an outcome. Each exposure is modelled to have a true continuous effect on the outcome but is only observed to take one of three categories (0/1/2) to reflect the setup of our body size data. For each exposure a set of 150 SNPs are available which predict the exposures. The model is estimated using a two-sample multivariable MR estimation where the SNP exposure associations are estimated in the same sample for both exposures and the SNP outcome association is estimated in a separate sample.

Within this model we investigated five misclassification scenarios; (1) no misclassification in either exposure, (2) random misclassification of 15% of the data in exposure 1 (the equivalent of early life body size), (3) random misclassification of 30% of the data in exposure 1, (4) misclassification of 30% of the data in exposure 1 to the level observed for exposure 2 (the equivalent of adult body size) and (5) misclassification of 30% of the data in exposure 1 to one category lower than the true value. Setting 4 represents a scenario where individuals misremember their childhood BMI as being the same (relative to others) as their current body size and setting 5 represents a

misclassification where respondents remember their childhood body size as being lower than it was. In each case we considered three scenarios; where only exposure 2 has an effect on the outcome, where exposures 1 and 2 both have positive effects on the outcome and where exposure1 has a negative effect on the outcome and exposure 2 has a positive effect on the outcome. In all cases exposure 2 is also more strongly predicted by the set of SNPs than exposure 1.

As the true effect of each exposure on the outcome is the effect of a change in the continuous variable underlying the observed categorical exposure we have calculated the effect on the outcome we expect to observe in the data as the effect of moving from the mean of one category to the mean of the next[1].

The results from the simulations are given in Supplementary Table 1. These results show that there is some bias in the estimated effects, due to the classification of the exposure into categories. This bias moves the estimated effect of both exposures away from the null. Random misclassification in X_1 or reclassification of X_1 to a lower category group weakens the strength of the instruments for X_1 and increases the bias in the estimated effect of X1, but does not affect the estimate for X_2. However, the estimated effect of X_2 is decreased when observations for X_1 are reclassified to the level observed for X_2 and there is a true causal effect of the X_1 on the outcome that is in the same direction as for X_2. When X_1 and X_2 have effects that act in opposite directions then each effect is biased away from the null. These results indicate that there is some potential for bias in the multivariable MR estimation when there is a true causal effect of X_1 on the outcome and misclassification in X_1 is dependent on X_2, however it is not possible to determine which direction such a bias would act in. The direction of the bias will depend on the association of each exposure with the outcome.

	No Misclassification			With Misclassification											
				15% Random			30% Random			30% X_2 Dependent			30% X_1 Dependent		
$\boldsymbol{\beta_1}$	**0**	**0.49**	**-0.49**	**0**	**0.49**	**-0.49**	**0**	**0.49**	**-0.49**	**0**	**0.49**	**-0.49**	**0**	**0.49**	**-0.49**
$\hat{\beta}_1$	-0.01	0.56	-0.58	-0.01	0.65	-0.67	-0.01	0.76	-0.79	-0.01	0.79	-0.81	-0.01	0.65	-0.67
Std. error	*0.03*	*0.04*	*0.03*	*0.03*	*0.04*	*0.03*	*0.04*	*0.05*	*0.04*	*0.04*	*0.05*	*0.04*	*0.03*	*0.04*	*0.03*
F – statistic	21.29	21.26	21.26	14.9	14.9	14.90	9.98	9.97	9.97	13.86	13.86	13.86	14.92	14.92	14.92
$\boldsymbol{\beta_2}$	**0.5**	**0.5**	**0.5**	**0.5**	**0.5**	**0.5**	**0.5**	**0.5**	**0.5**	**0.5**	**0.5**	**0.5**	**0.5**	**0.5**	**0.5**
$\hat{\beta}_2$	0.60	0.59	0.61	0.6	0.59	0.61	0.6	0.59	0.61	0.60	0.36	0.85	0.60	0.59	0.61
Std. error	*0.03*	*0.03*	*0.02*	*0.03*	*0.03*	*0.02*	*0.03*	*0.03*	*0.25*	*0.03*	*0.03*	*0.03*	*0.03*	*0.03*	*0.02*
F – statistic	28.29	28.31	28.31	28.29	28.29	28.29	28.23	28.27	28.26	30.79	30.79	30.79	28.3	28.3	28.3

N = 50,000. 2000 repetitions. Random misclassification is introduced by randomly re-allocating 15% or 30% of the observations for X_1 to a category (0/1/2), with 5 or 10 % allocated to each category respectively. X_2 Dependent misclassification is introduced by re-allocating 30% of observations for X_1 to their observed category for X_2. X_1 dependent misclassification is introduced by re-allocating 30% of the observations to the category lower (with observations in the lowest category left unchanged).

1. Greenland S, Longnecker MP. Methods for trend estimation from summarized dose-response data, with applications to meta-analysis. *Am J Epidemiol* 1992;135(11):1301-9. doi: 10.1093/oxfordjournals.aje.a116237 [published Online First: 1992/06/01]
